# Association of War With Vaccination Dropout Among Children Younger Than 2 Years in the North Wollo Zone, Ethiopia

**DOI:** 10.1001/jamanetworkopen.2022.55098

**Published:** 2023-02-07

**Authors:** Muluemebet Kassa Mezen, Getasew Assefa Lemlem, Yemisrach Belete Biru, Abebaw Mengesha Yimer

**Affiliations:** 1 School of Public Health, College of Health Science, Woldia University, Woldia, Ethiopia; 2 Department of Statistics, Faculty of Natural and Computational Science, Woldia University, Woldia, Ethiopia; 3 School of Public Health, College of Health Science, Woldia University, Woldia, Ethiopia; 4 Department of English Language and Literature, Faculty of Social Science, Woldia University, Woldia, Ethiopia

## Abstract

**Importance:**

A civil war that lasted for about 6 months in the North Wollo zone of Ethiopia destroyed numerous health care facilities. However, no studies have been conducted to determine the association of the war with vaccination dropout in the area.

**Objective:**

To assess the association of war with vaccination dropout among children younger than 2 years in the North Wollo zone.

**Design, Setting, and Participants:**

This population-based cross-sectional study was conducted in the North Wollo zone from April 1 to June 30, 2022. Participants included children younger than 2 years and their mothers. A multistage sampling technique was used to select the participants.

**Main Outcomes and Measures:**

A vaccination dropout rate (yes or no) was assessed by interviewing mothers.

**Results:**

The study included 449 children younger than 2 years and their mothers, 291 (64.8%) of whom were 20 to 34 years of age. Almost all of the mothers (426 [94.9%]) were married. More than half of the mothers (271 [60.4%]) had a primary level education. Seventy-one children (15.8%) in the area received all basic vaccinations. One hundred ninety-eight children (44.1%) who started vaccination prior to the war dropped out of the immunization program. Additionally, 64 children (14.3%) born during the war did not receive any vaccination. Losing a family member (adjusted odds ratio [AOR], 3.11 [95% CI, 1.63-5.93]; *P* = .001), not being informed about catch-up vaccination (AOR, 2.18 [95% CI, 1.39-3.43]; *P* < .001), being a rural resident (AOR, 2.22 [95% CI, 1.37-3.58]; *P* < .001), home birth (AOR, 1.75 [95% CI, 1.11-2.77]; *P* = .002), and length of war (AOR for 5 months, 0.51 [95% CI, 0.28-0.93; *P* = .04]) were associated with the outcome variable.

**Conclusions and Relevance:**

The findings of this cross-sectional study suggest that nearly 60% of children in the North Wollo zone remained undervaccinated or unvaccinated. Stakeholders should make coordinated efforts to overcome the humanitarian crisis in the area and optimize the accessibility of health services.

## Key Points

**Question:**

Is there an association between vaccination dropout among children younger than 2 years and war?

**Findings:**

In this cross-sectional study that included 449 children younger than 2 years and their mothers in the North Wollo zone of Ethiopia, the proportions of children who were undervaccinated and unvaccinated were 44% and 14%, respectively. Logistic regression showed an association between war and vaccination dropout.

**Meaning:**

The findings of this study suggest that restoring vaccination programs may be warranted to prevent the emergence of infectious diseases.

## Teaser

This cross-sectional study assesses the association of war with vaccination dropout among children younger than 2 years in the North Wollo zone of Ethiopia.

## Introduction

Vaccine-preventable diseases (VPDs) have been shown to cause 25% of deaths among children younger than 5 years worldwide and 50% among children younger than 5 years in Africa and Southeast Asia.^1,2,3^ An expanded program on immunization is one of the most crucial and efficient public health interventions designed by World Health Organization to prevent and eradicate VPDs.^4,5^ Since the establishment of this program in 1974, the morbidity and mortality of children younger than 5 years due to VPDs has been greatly reduced.^6,7^ It is estimated that up to 3 million deaths of children annually are prevented due to childhood immunization.^1^ For a child to be considered as completely or fully immunized, within 1 year of life, he or she has to receive a bacille Calmette-Guérin (BCG) vaccination against tuberculosis; 3 doses of pentavalent vaccine to prevent diphtheria, pertussis, tetanus, hepatitis B, and *Haemophilus influenzae* type B; at least 3 doses of polio vaccine; and 1 dose of measles vaccine.^8^ In 2021, 81% of infants worldwide received 3 doses of the vaccine protective against diphtheria, pertussis, and tetanus.^9^ According to the latest national survey, 44% of children in Ethiopia received basic vaccinations at some point in their lives; 40% of these children received the basic vaccines while they were infants.^10^ Despite increasing immunization coverage, a significant number of children are still missing out on these life-saving vaccines.

A vaccination dropout rate is the rate difference between the initial vaccine and the last vaccine.^11^ It is used as an indicator to evaluate the performance of immunization programs. In 2021, it is estimated that about 25 million infants were unvaccinated or undervaccinated; 60% live in 10 countries, including Ethiopia.^9^ In 2019, 39% of children in Ethiopia did not receive the third diphtheria, pertussis, and tetanus vaccine.^10^ Studies showed that counseling mothers about vaccinations, fear of adverse effects of vaccines, use of postnatal care, not receiving a tetanus toxoid vaccine, poor maternal knowledge about vaccine, mother’s educational status, long waiting hours at health facilities, marital status, births without skilled birth attendants, place of residence, and living further than 30 minutes from the nearest vaccination facility can lead to vaccination dropout. In conflict areas, however, security-related factors are the major determinants of immunization coverage.^11,12,13,14,15^

War poses a challenge to human health and the health care system and is one of the top 10 causes of death in the world.^16,17^ Disruption of basic health services, displacement, and food shortage due to war can exacerbate the incidence of infectious diseases.^18^ The civil war in northern Ethiopia began in November 2020 and spread to the Amhara region in July 2021. The North Wollo zone of the Amhara region was heavily exposed to the escalated conflict in the area; the conflict in the North Wollo zone began in July 2021 and continued to December 2021. As a result of the conflict, more than 50% of health care facilities in the Amhara region were damaged, and maternal and child health services were interrupted for more than 70 000 pregnant and lactating women.^19,20,21^ Immunization service was no exception. Previous studies showed that vaccination service delivery declined in conflict areas due to security problems, displacement of health care workers, destruction of health care facilities, scarcity of resources, and other emerging issues.^18,19^ However, the association of war with vaccination dropout was not assessed in the study area. Therefore, the objective of this study was to assess the association of war with vaccination dropout among children younger than 2 years in the North Wollo zone of Ethiopia.

## Methods

### Study Design and Setting

We conducted a community-based cross-sectional study from April 1 to June 30, 2022, in the North Wollo zone of Ethiopia. This study was approved by the Woldia University Research Ethics Review Committee. Before the data collection of the study was initiated, the data collectors explained the objectives of the study. They also clarified that participation in the study was voluntary and that participant confidentiality would be maintained. Following this, verbal consent was obtained from the mothers. The respondents’ confidentiality was assured by excluding names and identifiers from the questionnaire. This study followed the Strengthening the Reporting of Observational Studies in Epidemiology (STROBE) reporting guideline.

### Sample Size Determination and Sampling Techniques

A single population proportion formula was used to determine the sample size of the study. The proportion of children who returned to immunization after the war took place was 25.8%,^2^ the nonresponse rate was 5%, and the design effect was 1.5. This gives a total sample size of 463 participants.

A multistage sampling technique was used to select the study participants. First, 4 *woredas*, or districts (Woldia, Harbu, Dawunt, and Meket), were selected randomly from the 14 *woredas* in the North Wollo zone. Then, 2 *kebeles*, or villages, were selected randomly from each of the selected woredas (*Kebele* 01 and 05 from Woldia, *Kebele* 01 and 06 from Habru, *Kebele* 02 and 03 from Dawunt, and *Kebele* 01 and 04 from Meket). After this, the lists of households with children younger than 2 years were prepared in collaboration with the respective *kebele* leaders and health extension workers. Finally, the data were collected at every 10 households using systematic random sampling. Children whose mothers were critically ill were excluded.

### Study Variables

The outcome of this study was vaccination dropout (yes or no). The independent variables were sociodemographic factors (age of mother, mother’s occupational status, marital status, mother’s educational status, residence, religion, family size, number of children younger than 5 years, parity, and sex of the child), war-related factors (food security, loss of a family member due to the war, family displaced due to war, and the length of war in the area), and factors related to maternal use of health services (antenatal care [ANC] during the last pregnancy, being counseled about vaccination, ever being informed about catch-up vaccination, and place of childbirth).

### Definition of Terms

*Vaccination dropout* indicated a child who started vaccination before the war (July 2021) and did not return after the war (December 2021), or a newborn who was scheduled to be vaccinated during the war but was not vaccinated. *Fully vaccinated* indicated a child who received 1 dose of BCG vaccine, 3 doses of pentavalent vaccine, 3 doses of polio vaccine, and 1 dose of measles vaccine within the first year of life.^22^ *Defaulter* indicated a child who received at least 1 dose of vaccine but not all doses.^23^ *Catch-up immunization* indicated the practice of giving a vaccine to a child who failed to receive it at the recommended age. Catch-up vaccines may be given to a child who has not been previously vaccinated, who has missed a scheduled vaccine dose, or who has not completed vaccinations.^23^

### Data Quality Control

The data were collected from the mothers using a structured, pretested, interviewer-administered questionnaire. Before the initiation of the data collection, training was given to data collectors and supervisors on the data collection process. To increase the quality of the data collection tool, a pretest was conducted on 5% of the sample size in the town of Hara in the Amhara region.

### Statistical Analysis

After the data were collected using KoboToolbox (Kobo), they were exported to SPSS, version 25 (IBM Corporation), for cleaning and analysis. Descriptive statistics were conducted to describe the findings. Logistic regression was conducted to determine the factors associated with vaccination dropout. Variables with a 2-sided *P* value of less than .05 during binary logistic regression analysis were exported to a multivariable logistic regression. We used odds ratios (ORs) to determine the presence of the association, and a 2-sided *P* value of less than .05 was used to declare a statistical significance during multiple logistic regression analysis. There were no missing values during the analysis.

## Results

### Sociodemographic Characteristics of Study Participants

Of the 463 mothers approached, a total of 449 mothers with their children were included in the final analysis, for a response rate of 97.0%. Among these, 291 (64.8%) were aged 20 to 34 years, and almost all of the respondents (426 [94.9%]) were married. Regarding the educational status of the mothers, more than half (271 [60.4%]) had a primary level education. Around two-thirds of the respondents (281 [62.6%]) resided in rural areas (Table 1).

**Table 1.** Sociodemographic Characteristics of Study Participants

Characteristic	No. (%)^a^
Age of mother, y	
<20	43 (9.6)
20-34	291 (64.8)
≥35	115 (25.6)
Age of child, mo	
6-11	209 (46.5)
12-23	240 (53.5)
Sex of child	
Boys	234 (52.1)
Girls	215 (47.9)
Marital status of mother	
Married	426 (94.9)
Not married	23 (5.1)
Educational status of mother	
None	66 (14.7)
Elementary level	271 (60.4)
Secondary level	58 (12.9)
Above secondary level	54 (12.0)
Educational status of father	
None	41 (9.1)
Elementary level	193 (43.0)
Secondary level	137 (30.5)
Above secondary level	78 (17.4)
Occupation of mother	
Housewife	221 (49.2)
Farmer	147 (32.7)
Other	81 (18.0)
Place of residence	
Urban	168 (37.4)
Rural	281 (62.6)
No. of family members	
<5	233 (51.9)
≥5	216 (48.1)
No. of children aged <5 y in family	
1	273 (60.8)
2	152 (33.9)
3	24 (5.3)

^a^ Data are reported as No. (%) of 449 mothers or 449 children as indicated. Percentages have been rounded and may not total 100.

### Use of Maternal Health Services by Mothers

A total of 366 mothers (81.5%) had ANC follow-up during their last pregnancy, of whom 227 (62.0%) received the tetanus toxoid vaccine. More than one-third of the mothers (164 [36.5%]) gave birth at home during their last pregnancy. More than half of the mothers with ANC follow-up (187 of 366 [51.1%]) did not get counseling on vaccination, and about half (226 [50.3%]) were informed about catch-up vaccinations. More than two-thirds of the mothers (294 [65.5%]) had to walk for 30 minutes to 1 hour to reach the nearest health facility and only 157 (35.0%) replied that the nearest health facility had an alternative power source (Table 2).

**Table 2.** Maternal Use of Health Care Services Among the Study Participants

Characteristic	No. (%) of mothers (N = 449)
ANC during last pregnancy	
Yes	366 (81.5)
No	83 (18.5)
Counseled about vaccination during ANC^a^	
Yes	179 (48.9)
No	187 (51.1)
Place of birth for the last pregnancy	
Home	164 (36.5)
Health care facility	285 (63.5)
Informed about catch-up vaccination	
No	223 (49.7)
Yes	226 (50.3)
Received tetanus toxoid vaccine during last pregnancy^a^	
Yes	227 (62.0)
No	139 (38.0)
Distance from health care facility	
<30 min	146 (32.5)
30 min to 1 h	294 (65.5)
>1 h	9 (2.0)
Child returns to the immunization program	
Yes	251 (55.9)
No	198 (44.1)
Nearest health facility has alternative power source	
No	292 (65.0)
Yes	157 (35.0)

Abbreviation: ANC, antenatal care.

^a^ Includes the 366 women who received ANC.

### War-Related Factors

More than half of the mothers who participated in the study (258 [57.5%]) stated that they had faced food insecurity because of the war. Sixty-four respondents (14.3%) mentioned that they lost at least 1 family member due to the war and 239 (53.2%) were temporarily displaced to other areas due the war (Table 3).

**Table 3.** War-Related Factors of the Study Participants

Characteristic	No. (%) (N = 449)
Household food security	
Secure	191 (42.5)
Insecure	258 (57.5)
Lost family members due to the war	
Yes	64 (14.3)
No	385 (85.7)
Family displaced due to war	
Yes	239 (53.2)
No	210 (46.8)
Duration of war, mo	
1	153 (34.1)
5	222 (49.4)
6	74 (16.5)

### Prevalence of Vaccination Dropout

In the present study, 71 children younger than 2 years (15.8%) received all of the basic vaccinations. At the time of the survey, 73 of 449 children (16.3%) received the first vaccine dose (BCG, pentavalent, and polio), 78 (17.4%) received the second vaccine dose (pentavalent and polio), and 168 (37.4%) received the third vaccine dose (pentavalent and polio). A total of 198 children (44.1%) failed to return to the immunization program after the war and 64 (14.3%) who were born during the war remained unvaccinated.

### Association of War With Vaccination Dropout

Binary and multiple logistic regression was conducted to determine factors associated with vaccination dropout among children younger than 2 years. In multivariable logistic regression analysis, place of childbirth during the last pregnancy, being informed about catch-up vaccine, place of residence, length of the war in the area, and losing a family member due to the war were associated with vaccination dropout.

Losing a family member due to the war tripled the risk of vaccination dropout (adjusted OR [AOR], 3.11 [95% CI, 1.63-5.93]; *P* = .001). The odds of vaccination dropout were twice as high among children living in rural areas compared with children living in urban areas (AOR, 2.22 [95% CI, 1.37-3.58]; *P* < .001). Lack of information on catch-up vaccinations was associated with higher vaccination dropout (AOR, 2.18 [95% CI, 1.39-3.43]; *P* < .001). Compared with giving birth in health care facilities, home delivery increased the risk of vaccination dropout by 75% (AOR, 1.75 [95% CI, 1.11-2.77]; *P* = .002). On the other hand, vaccination dropout decreased by 80% (AOR, 0.20 [95% CI, 0.10-0.39]; *P* < .001) and 49% (AOR, 0.51 [95% CI, 0.28-0.93]; *P* = .04) in areas affected by war for 1 month and 5 months, respectively (Table 4). The fitness of the model was checked using the Hosmer-Lemeshow goodness-of-fit test (*P* = .29).

**Table 4.** Factors Associated With Vaccination Dropout Among Children Younger Than 2 Years in the North Wollo Zone of Ethiopia

Variables	COR (95% CI)	AOR (95% CI)	*P* value^a^
Family displaced due to war			
Yes	0.60 (0.42-0.89)	0.81 (0.52-1.27)	.12
No	1 [Reference]	1 [Reference]	NA
Household food security			
Insecure	1.77 (1.21-2.59)	1.39 (0.90-2.15)	.06
Secured	1 [Reference]	1 [Reference]	NA
Place of birth for the last pregnancy			
Home	2.43 (1.64-3.60)	1.75 (1.11-2.77)	.002
Health facility	1 [Reference]	1 [Reference]	NA
Did you hear about catch-up vaccination?			
No	2.89 (1.96-4.25)	2.18 (1.39-3.43)	<.001
Yes	1 [Reference]	1 [Reference]	NA
Place of residence			
Rural	2.52 (1.68-3.77)	2.22 (1.37-3.58)	<.001
Urban	1 [Reference]	1 [Reference]	NA
Length of war, mo			
1	0.23 (0.12-0.15)	0.20 (0.10-0.39)	<.001
5	0.54 (0.32-0.94)	0.51 (0.28-0.93)	.04
6	1 [Reference]	1 [Reference]	NA
Lost family member due to war			
Yes	2.31 (1.39-4.13)	3.11 (1.30-5.93)	.001
No	1 [Reference]	1 [Reference]	NA
Educational status of mother			
None	1.80 (0.81-4.01)	0.76 (0.31-1.87)	.85
Primary level	3.37 (1.73-6.57)	1.25 (0.59-2.64)	.49
Secondary level	1.79 (0.79-4.08)	1.06 (0.43-2.63)	.53
Above secondary level	1 [Reference]	1 [Reference]	NA

Abbreviations: AOR, adjusted odds ratio; COR, crude odds ratio; NA, not applicable.

^a^ Hosmer-Lemeshow test *P* = .29.

## Discussion

The North Wollo zone was under civil war for approximately 6 months. Therefore, the present study was conducted to assess the association of war with vaccination dropout among children younger than 2 years in the study area. The study showed that only 15.8% of the children were able to complete their vaccination schedule. On the other hand, 44.1% of the children who started vaccination before the onset of the war were unable to adhere to their vaccination schedule. Also, 14.3% of the children born during the war were unvaccinated. Place of birth, loss of family member due to the war, being informed about catch-up vaccination, place of residence, and length of war were found to be associated with the outcome variable.

In this study, the vaccination dropout rate among children younger than 2 years in the North Wollo zone was 44.1%. This finding was much higher than those of studies conducted in war-free areas such as Ghana and southwest Ethiopia (5.5% and 25.8%, respectively).^2,24^ This result is also higher than the dropout rates of other countries as stated in global routine vaccine coverage in 2018.^25^ However, the result is supported by other studies conducted in northwest Ethiopia^15^ and another 16 countries.^26^ This shows that war can adversely affect immunization coverage. This could be caused by decreasing the availability and accessibility of health services as well as creating other emergencies.

In the present study, losing a family member due to the war tripled the likelihood of vaccination dropout. This finding is also supported by studies conducted in Yemen and Syria that noted a negative link between conflict and the use of health services.^27,28^ This could be due to the fact that losing a family member may impose economical, social, and psychological burdens that prevent individuals from using preventive health services like immunization.

Residing in rural areas doubles the risk of vaccination dropout. This finding was in accordance with the findings of other studies conducted in northwest Ethiopia,^15^ Nepal,^29^ and Shewa Robit.^30^ This could be due to the fact that health services are inaccessible in rural areas compared with urban areas. Moreover, it might take a longer time to rehabilitate and reconstruct the health system in rural areas than in urban areas.

Mothers who were never informed about catch-up vaccination were twice as likely to drop out of immunization than mothers who had information about catch-up vaccination. This finding was in line with studies conducted in the Gondar zone^11^ and other parts of Ethiopia, including the Hadiya zone.^31,32^ This might be due to the fact that mothers who have information on catch-up vaccination most likely get their children vaccinated even if they missed the regular schedule.

Home delivery increases the risk of dropping out of immunization by 75%. This finding is also supported by studies conducted in Nepal^29^ and western Ethiopia.^2^ The possible explanation could be mothers who give birth at home are more likely to have limited communication with health care professionals and information about immunization.

The present study showed that the length of the war in the study area was inversely associated with vaccination dropout. Vaccination dropout decreased by 80% and 49% in areas occupied by war for 1 month and 5 months, respectively. This might be because the effects (humanitarian, psychological, and economical) of the war might increase as the length of the war is prolonged.

### Strengths and Limitations

A strength of this study is that the findings can be generalized given that it was a community-based study. Additionally, it highlights the negative outcomes of a war that received little media coverage. As a limitation, a temporal relationship could not be established due to the nature of the study design. Additionally, since most of the mothers lost the vaccination charts, we could not confirm their responses. However, this is unlikely to affect the findings of the study, as different studies reported that maternal recall of a child vaccination is valid.^33,34,35,36^

## Conclusions

The findings of this cross-sectional study suggest that nearly 60% of children residing in the North Wollo zone of Ethiopia remain undervaccinated or unvaccinated. Place of childbirth, being informed about catch-up vaccination, place of residence, length of the war in the area, and loss of family members due to war were associated with vaccination dropout. Ethiopia was able to improve maternal and child health using the health extension program^37^ and the Women’s Development Army.^38^ Therefore, these programs should continue and strengthen to increase institutional delivery. These programs should also be used to inform mothers about catch-up vaccinations and to vaccinate home-born children. Health care professionals should create awareness of catch-up vaccinations. Stakeholders should also use the already existing community structures to promote health information. Moreover, stakeholders should make efforts to address cultural and structural challenges that reduce maternal use of health care services. Additional efforts should be made to alleviate the humanitarian crisis in the area and to rehabilitate the destroyed health facilities and infrastructures both at international and national levels, and vaccination campaigns should be prepared to increase vaccination coverage in the area.

## Notes

Supplement.
Data Sharing Statement
