# Supplementary material for: Association of War With Vaccination Dropout Among Children Younger Than 2 Years in the North Wollo Zone, Ethiopia
Source: JAMA Netw Open. 2023 Feb 7;6(2):e2255098. doi: 10.1001/jamanetworkopen.2022.55098 (PMC10408260; doi:10.1001/jamanetworkopen.2022.55098)
Supplement: Supplement. — Data Sharing Statement [file jamanetwopen-e2255098-s001.pdf]

Data Sharing Statement

Mezen. Association of War With Vaccination Dropout Among Children Younger Than 2 Years in the North Wollo Zone, Ethiopia. *JAMA Netw Open*. Published February 07, 2023. doi:10.1001/jamanetworkopen.2022.55098

Data
Data available: Yes
Data types: Deidentified participant data
How to access data: The deidentified data will be available from the corresponding author up on a reasonable request using the following email: getasew1@gmail.com
When available: With publication

Supporting Documents
Document types: None

Additional Information
Who can access the data: The data will be available for researchers whose proposed use of the data has been approved.
Types of analyses: The data will be made available for any type of analysis
Mechanisms of data availability: The data will be made available after the approval of a proposal
